# Safety, Efficacy, and Mechanistic Studies Regarding Citrus aurantium (Bitter Orange) Extract and *p*‐Synephrine

**DOI:** 10.1002/ptr.5879

**Published:** 2017-07-28

**Authors:** Sidney J. Stohs

**Affiliations:** ^1^ Creighton University Medical Center Kitsto Consulting LLC Frisco TX USA

**Keywords:** *p*‐synephrine, bitter orange extract, Citrus aurantium, mechanisms, cardiovascular effects, receptor binding, efficacy

## Abstract

Citrus aurantium L. (bitter orange) extracts that contain p‐synephrine as the primary protoalkaloid are widely used for weight loss/weight management, sports performance, appetite control, energy, and mental focus and cognition. Questions have been raised about the safety of p‐synephrine because it has some structural similarity to ephedrine. This review focuses on current human, animal, in vitro, and mechanistic studies that address the safety, efficacy, and mechanisms of action of bitter orange extracts and p‐synephrine. Numerous studies have been conducted with respect to p‐synephrine and bitter orange extract because ephedra and ephedrine were banned from use in dietary supplements in 2004. Approximately 30 human studies indicate that p‐synephrine and bitter orange extracts do not result in cardiovascular effects and do not act as stimulants at commonly used doses. Mechanistic studies suggest that p‐synephrine exerts its effects through multiple actions, which are discussed. Because p‐synephrine exhibits greater adrenergic receptor binding in rodents than humans, data from animals cannot be directly extrapolated to humans. This review, as well as several other assessments published in recent years, has concluded that bitter orange extract and p‐synephrine are safe for use in dietary supplements and foods at the commonly used doses. Copyright © 2017 The Authors Phytotherapy Research Published by John Wiley & Sons Ltd.

## Introduction

This review focuses on current human, animal, and *in vitro* studies that address the safety, efficacy, and mechanisms of action of bitter orange (Citrus aurantium *L.*) extracts and their primary active constituent *p*‐synephrine. Bitter orange extracts are aqueous/ethanolic extracts of the dried immature fruits of C. aurantium that are harvested in May and June. In China, the immature fruits of bitter orange are also known as *Fructus aurantii immaturus* (Chen and Chen, 2004). Bitter orange extracts have been used as dietary supplements for approximately 20 years for weight management, energy production, and sports performance, as well as appetite control and energy.

In traditional Chinese medicine, the peel and/or whole dried immature fruit of C. aurantium (bitter orange) has been used for hundreds of years for a variety of health applications, including indigestion, diarrhea and dysentery, constipation, and as an expectorant (Chen and Chen, 2004; Fang *et al*., 2009; Xutian *et al*., 2014). Bitter orange has been used in South American folk medicine to treat insomnia, anxiety, and epilepsy (Pimenta *et al*., 2016). Bitter orange is also known as Seville orange because it has been grown in Seville Spain for over 800 years where it is used in various food products including marmalades, syrups, and juices that are widely distributed and consumed (Stohs and Preuss, 2011a; Stohs *et al*., 2011a).

**Figure 1:**
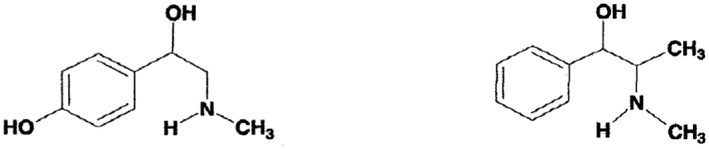
Structures of *p*‐synephrine and ephedrine.

Bitter orange extracts contain *p‐*synephrine, which comprises about 90% or more of the total protoalkaloids, and to which bitter orange extracts are standardized (Stohs and Preuss, 2011a; Stohs *et al*., 2011a, 2012; Stohs and Shara, 2013; Carpene *et al*., 2014). *p*‐Synephrine is a phenylethylamine derivative with a hydroxyl group in the *para* position on the benzene ring of the molecule and has some structural similarity to ephedrine (Fig. 1). However, ephedrine is a phenylpropylamine derivative and does not contain a substituted hydroxyl group on the phenyl (benzene) ring.

Questions have been raised regarding the safety of *p*‐synephrine because it has some structural similarity to ephedrine, and *p*‐synephrine is widely referred to as a stimulant and is assumed to exhibit cardiovascular activity because of its structural similarity to ephedrine (see, for example, Penzak *et al*., 2001; Bent *et al*., 2004; Fugh‐Berman and Myers, 2004; Haaz *et al*., 2010, Anon, 2010; Inchiosa, 2010; Rasmussen *et al*., 2012; Natural Medicines Comprehensive Database, 2016; OPPS, 2017; Bakhiya *et al*., 2017). However, the chemical differences between *p‐*synephrine and ephedrine greatly alter the stereochemistry, pharmacokinetic, adrenergic receptor binding, and physiological/pharmacological properties (Stohs and Preuss, 2011b; Stohs *et al*., 2011b). Therefore, the effects observed with ephedrine cannot be extrapolated to *p*‐synephrine and bitter orange extracts. As will be discussed, *p*‐synephrine is without the adverse cardiovascular effects associated with ephedrine at commonly used doses.

There are structural similarities between *p*‐synephrine and the catecholamines epinephrine and norepinephrine that contain hydroxyl groups at both the *meta* and *para* positions of the benzene ring. Several studies have concluded that it is the hydroxyl group in the *meta* position of the ring that primarily promotes adrenergic receptor binding and the subsequent cardiovascular effects, while the single hydroxyl group in the *para* position, as in the case of *p*‐synephrine, decreases adrenergic receptor binding (Mukherjee *et al*., 1976; Ma *et al*., 2010). Therefore, cardiovascular effects observed with epinephrine and norepinephrine cannot be directly extrapolated to *p*‐synephrine.

This review will summarize the observations and conclusions of several previous reviews and focus on studies published because these reviews deal with human studies and animal studies as well as *in vitro* studies that provide information concerning safety, efficacy, and the mechanisms of action of *p*‐synephrine and bitter orange extracts. Also discussed are the absorption and metabolism of *p*‐synephrine.

## Summary of Previous Reviews

Several reviews summarized approximately 20 published and unpublished clinical studies involving about 360 subjects who received a product containing a bitter orange extract and *p*‐synephrine alone or in combination with other ingredients (Marles, 2011; Stohs *et al*., 2012; Stohs and Shara, 2013; Lynch, 2013). Over 200 of the subjects in these studies were reported to be overweight/obese, approximately 200 of the subjects consumed caffeine (up to 528 mg/day) in combination with *p*‐synephrine (up to 80 mg/day) as part of the study, while 160 of the subjects summarized in these reviews consumed *p*‐synephrine (bitter orange extract) alone (Stohs *et al*., 2012; Stohs and Shara, 2013).

Regarding potential cardiovascular effects, small increases in diastolic effects were reported for a total of 25 subjects in two studies (Bui *et al*., 2006; Haller *et al*., 2008) with no increases reported for 335 subjects (21 studies). A small (4 beats/min) increase in heart rate was reported in one study involving 15 subjects at a dose of 50 mg *p*‐synephrine but not confirmed by any other studies at doses up to 80 mg (Bui *et al*., 2006). The results are only discussed relative to placebo. As compared with baseline, small clinically insignificant increases in heart rate (2.4 beats/min) and systolic blood pressure (4.5 mm Hg) with a decrease in diastolic blood pressure (2.0 mm Hg) occurred. These results have not been replicated by other studies.

With respect to the study by Haller *et al*. (2008) involving ten subjects, a meal given at 3 h after ingestion of 46.9‐mg *p*‐synephrine resulted in an increase in heart rate, which was misinterpreted as being due to *p*‐synephrine at 6 h after dosing (Stohs *et al*., 2012; Stohs and Shara, 2013).

An extensive 49‐page review and health risk assessment of *p*‐synephrine, *p*‐octopamine, and caffeine was conducted by the Natural Health Products Directorate of Health Canada, which also defined guidelines for use of these ingredients (Marles, 2011). The report concluded that consumption of up to 50 mg per day of *p*‐synephrine alone in healthy adults ‘is not likely to cause any adverse health consequences’. The report similarly concluded that use of products containing 40 mg per day or less of *p*‐synephrine in combination with 320 mg per day or less of caffeine was also not likely to cause adverse effects. The guidelines for consumption of *p*‐octopamine were identical to *p*‐synephrine. Health Canada is comparable to the US Food and Drug Administration (FDA). This report by Health Canada was prepared before a number of studies described in the succeeding texts were published, which have demonstrated safety at higher levels. Unfortunately, this report has not been widely distributed because it was not concurrently translated into French.

Intertek‐Cantox provides scientific, toxicology, and regulatory services with respect to dietary supplements. Its reports are used as a basis for making recommendations regarding the use and safety of supplements. Intertek‐Cantox conducted an in depth scientific literature review and issued a report on the safety of bitter orange extract (*p*‐synephrine) alone and in combination with caffeine and provided recommended guidelines for their use (Lynch, 2013). The report concluded that ‘*p*‐synephrine is unlikely to have significant effects on inotropy, vasoconstriction, or blood pressure’, and noted that ‘the use of *p*‐synephrine alone or in combination with caffeine, within the specified limits, is not considered to pose significant concerns’ (Lynch, 2013).

The Intertek‐Cantox Report indicated that the following specified dosage limits were ‘not likely to cause adverse effects’, which included 70 mg of *p*‐synephrine alone or 40 mg in combination with 320 mg of caffeine. Furthermore, if taken as divided doses spaced out over the course of the day, 100 mg of *p*‐synephrine alone or 70 mg *p*‐synephrine in combination with 400 mg caffeine is unlikely to be associated with adverse health effects. As was the case with the Health Canada Report (Marles, 2011), the Intertek‐Cantox Report (Lynch, 2013) was also published before a number of more recent studies described in the succeeding texts were published that demonstrated safety at higher doses.

Stohs (2010) previously reviewed 22 US FDA adverse event reports (AERs) from April 2004 through October 2009, which involved bitter orange (C. aurantium)‐containing products, as well as 10 clinical case reports published during this same time interval concerning the possible involvement of bitter orange *(p*‐synephrine)‐containing products with various adverse events. Bitter orange extract and/or *p*‐synephrine were implicated as the probable causative agent in the case reports by all of the authors in spite of the fact that, in all reported AERs and cases, the products contained multiple herbal ingredients.

The adverse events associated with the published clinical case studies included acute lateral‐wall myocardial infarction, exercise‐induced syncope associated with QT prolongation, vasospasm and stroke, ischemic stroke, ventricular fibrillation, variant angina, ischemic colitis, coronary spasm and thrombosis, and ST segment‐elevation myocardial infarction. In addition, in one case report, it was suggested that a bitter orange/*p*‐synephrine‐containing dietary supplement may have masked hypotension and bradycardia, while exacerbating weight loss in an individual with anorexia nervosa, although no evidence was presented that an adverse event had occurred (Stohs, 2010).

Each of the case studies specifically concluded that C. aurantium, bitter orange, or *p*‐synephrine was the most likely causative agent, although the products consumed were all poly‐herbal, poly‐alkaloidal, and poly‐protoalkaloidal. However, a wide range of confounding factors that were not apparently taken into consideration existed among the published case reports, including heart murmur, preexisting heart disease, hypertriglyceridemia, obesity, a history of smoking, gastroesophageal disease, physical inactivity, sickle cell trait, dehydration, pneumonia, possible use of anabolic steroids and/or performance‐enhancing drugs, high caffeine intake, and high alcohol consumption. Furthermore, products were not always being taken as recommended, and it was not always clear if the subjects were using other unreported dietary supplements and/or drugs (Stohs, 2010).

A case study that was not included in the review in the preceding texts was reported by Firenzuoli *et al*. (2005). The subject was reported to have consumed a product that contained 500 mg of a bitter orange extract (6% *p*‐synephrine) and subsequently experienced tachycardia. A repeat occurred a month later after consuming the dietary supplement. Unfortunately, the authors do not indicate the composition of the dietary supplement and what other ingredients were present. No analysis was conducted on the product in an attempt to identify ingredients that may have been responsible. As a consequence, it is not clear whether the response was due to the bitter orange extract or some other ingredient or adulterant that was present. It is also not clear whether this was a pharmacological/toxicological response or an allergic or hypersensitivity response to some constituent present in the product. Due to a lack of adequate information, it is not possible to conclude that the causative agent was the bitter orange extract and *p‐*synephrine.

Since the review of adverse events and case reports (Stohs, 2010), Doctorian and Do (2017) published a case on an ascending aortic dissection in a male who consumed two doses of a preworkout supplement of unidentified composition that was reported to contain *p*‐synephrine. In actuality, the product contained 10 mg of *p‐*synephrine, 135 mg of caffeine, 1.5 g of beta alanine, Mucuna pruriens extract standardized for l‐3,4‐dihydroxyphenylalanine (L‐DOPA), and other ingredients. The authors did not review the literature and cited speculative review articles and case reports. The authors did not know how much *p‐*synephrine was in the product and that no controlled studies have ever shown adverse cardiovascular effects associated with *p*‐synephrine. They did not discuss the fact that this amount of *p*‐synephrine is widely consumed on a daily basis by many individuals in citrus juices and other food products without adverse effects. It is not possible to show a relationship between the product and the ascending aortic dissection, much less a relationship with any ingredient including *p‐*synephrine. Caffeine is known to exhibit cardiovascular activity, while *p*‐synephrine is not.

Lude *et al*. (2016) reviewed plant food supplements associated with a large number of plants including C. aurantium involving human adverse case reports. These authors referenced most of the case studies reviewed in the preceding texts (Stohs, 2010) and cited review articles that made reference to the same case studies. They also cited a case of bronchospasm after handling orange flavedo (zest), which appears to be a sensitivity reaction most likely to the volatile oils present in the zest and is unrelated to bitter orange extract.

Although these case reports raise the level of awareness with regard to the use of complex herbal products, it is not possible to extrapolate the cause of these adverse effects to *p*‐synephrine, which may have been present in the products. In no case was the product analyzed to determine the actual composition of the product, or the amount of *p*‐synephrine present, if at all. No evidence was provided showing a direct link between bitter orange extract and the adverse events. In some cases, the actual composition of the product was not completely identified. In no case was a challenge test conducted to determine if the identified product was in fact associated with the described adverse event (Stohs, 2010; Stohs *et al*., 2012; Stohs and Shara, 2013).

Since the publication of the review regarding case studies and AERs (Stohs, 2010), few new case reports have been published, presumably due to an increased level of awareness of the poor quality of the previously published reports and a growing awareness of requirements for publishing case reports. Furthermore, the overall quality of dietary supplements may have improved with fewer contaminants or adulterants. It should be kept in mind that ‘case reports are incomplete, uncontrolled, retrospective, lack operational criteria for identifying when an adverse event has actually occurred, and resemble nothing so much as hearsay evidence, a type of evidence that is prohibited in all courts of industrialized societies’ (Karch, 2007).

## More Recent Human Studies

The following studies have been published, are in press, or have been submitted for publication since the reviews in the preceding texts were written and published. In a double‐blinded, placebo‐controlled safety study, 46 healthy human subjects were given a standardized bitter orange extract (Advantra Z^®^) in capsule form that contained 49 mg of *p*‐synephrine twice a day (total of 98 mg of *p*‐synephrine/day) for 60 days, while 23 subjects received the placebo (Kaats *et al*., 2013). No adverse effects were observed at the end of the 60 days with respect to heart rate, blood pressures, blood chemistries, or blood cell counts with differentials. No untoward effects were observed relative to cardiovascular, hepatic, renal, or hematopoietic systems. No differences were found between the *p*‐synephrine‐treated and the placebo groups, and no adverse events were reported by any of the subjects. This is the longest study with the highest dose of *p*‐synephrine that has been published to date, and no adverse effects were reported or observed under these conditions.

In a randomized, placebo‐controlled crossover designed study involving 12 male athletes, each subject was randomly assigned (in double‐blind manner) to a treatment sequence consisting of the use of three supplements in the form of two chews: (i) 100 mg of *p*‐synephrine (Advantra Z^®^); (ii) 100 mg of *p*‐synephrine with 100 mg of caffeine; and (iii) placebo (Ratamess *et al*., 2015; Ratamess *et al*., 2016). The supplements were consumed for 3 days. The subjects performed a controlled resistance exercise protocol. Each supplement treatment was separated by a 1‐week washout period. No adverse effects were observed or reported with respect to *p*‐synephrine consumption in the presence or absence of caffeine, while *p*‐synephrine increased desirable effects including lipolysis, energy expenditure, fat oxidation, and V0_2_ as well as carbohydrate mobilization. In addition, *p*‐synephrine increased total repetitions and volume load by approximately 10% without increasing blood lactate or ratings of perceived exertion. Consumption of caffeine in addition to *p*‐synephrine increased mean power and velocity of squat performance but had no additional effect on total repetitions and volume load (Ratamess *et al*., 2015).

A placebo‐controlled, randomized cross‐over study examined the effects of a standardized bitter orange extract (Advantra Z^®^) at a dose of 49 mg of *p*‐synephrine in capsule form on heart rate, blood pressure, electrocardiograms (ECGs), and blood chemistries in 18 male and female subjects over a 24‐h period of time (Shara *et al*., 2016). No significant changes occurred in ECGs, heart rates, blood pressures, serum chemistries, or blood cell counts, and no adverse effects were reported or observed. A small decrease in diastolic blood pressure (4.5 mm Hg) occurred at the 60‐min time point in the *p*‐synephrine‐treated group, contrary to what was observed in a small number of subjects (Bui *et al*., 2006; Haller *et al*., 2008). The authors concluded that *p‐*synephrine does not act as a stimulant at the dose used in the study.

In a follow‐up randomized, placebo‐controlled cross‐over study, bitter orange extract (Advantra Z^®^) at a dose of 49 mg of *p*‐synephrine daily in capsule form for 14 days to 16 male and female subjects was shown to have no significant effects on ECGs, heart rates, blood pressures, serum chemistries, or blood cell counts, and no adverse effects were reported or observed by any of the subjects (Stohs and Shara, 2013; Shara *et al*., 2017).

A review has summarized natural stimulant and nonstimulant thermogenic agents and discussed the controlling mechanisms involved in thermogenesis (Stohs and Badmaev, 2016). These authors have concluded that *p*‐synephrine, chlorogenic acid, forskolin, and capsaicin are nonstimulant thermogenic agents because they do not exhibit cardiovascular effects at commonly used doses as compared with caffeine and ephedrine, which are stimulant thermogenics.

In a randomized, double‐blind, cross‐over designed study,18 healthy subjects were given either a placebo or *p*‐synephrine HCl (3 mg/kg) orally, and energy expenditure and fat oxidation were measured at rest and during a cycle ergometer ramp exercise test (Gutierrez‐Hellin and Coso, 2016). Acute *p*‐synephrine HCl intake at rest had no effect on heart rate or blood pressure, nor did it have any effect on energy expenditure or substrate oxidation. However, during exercise at low‐to‐moderate intensities, *p*‐synephrine HCl increased the rate of fat oxidation while reducing the rate of carbohydrate oxidation.

Several factors must be taken into consideration with respect to this study (Gutierrez‐Hellin and Coso, 2016). Naturally occurring *p*‐synephrine exists in standardized bitter orange extracts in the *l*‐enantiomeric or [*R*‐(−)] enantiomeric form, whereas synthetic *p*‐synephrine HCl is a racemic mixture of the *l*‐enantiomeric and *d*‐enantiomeric forms and would be expected to exert less than half of the pharmacological activity of the naturally occurring *p*‐synephrine due to the fact that the *d*‐enantiomer exhibits about 100th the adrenergic receptor binding activity of the *l‐*enantiomer and is used as the HCl salt (Stohs *et al*., 2011b). Giving a dose of 3 mg of synthetic *p*‐synephrine HCl per kg represents a dose of 210 mg for a 70‐kg individual. The racemic mixture will have an activity of approximately 50% of the naturally occurring *l*‐enantiomeric or [R‐(−)] enantiomeric form, and because the synthetic *p‐*synephrine is used as the HCl salt, the 210 mg will be physiologically equivalent to approximately 86 mg of the naturally occurring *l‐*synephrine form of *p*‐synephrine present in bitter orange extracts.

In a study that assessed safety, energy, and appetite control, chocolate‐flavored chews containing 51.5 mg of *p*‐synephrine (Advantra Z^®^) or placebo were consumed 15–30 min before the two largest meals of the day for 15 or 30 days. Thus, the subjects consumed 103 mg of *p*‐synephrine or placebo daily. No changes in heart rate or blood pressure were noted, and no adverse effects were reported for either the *p*‐synephrine‐treated group (103 mg *p*‐synephrine per day) or the placebo control group. Statistically significant increases in energy and appetite/eating control were reported with respect to the *p*‐synephrine chew as compared with the placebo control chew (Kaats and Stohs, 2017). This study again demonstrated the absence of cardiovascular effects when consuming up to 103 mg of *p*‐synephrine per day.

The safety of a preworkout dietary supplement with and without *p*‐synephrine was examined (Jung *et al*., 2017). A preworkout supplement containing 270 mg of caffeine or the caffeine‐containing supplement with 20 mg of *p*‐synephrine was consumed daily for 8 weeks in a randomized, double‐blinded, placebo‐controlled manner involving 80 healthy men. Ingesting the preworkout supplement containing caffeine with and without *p*‐synephrine had no adverse effects on liver enzymes, kidney function, blood chemistries, or muscle enzymes relative to baseline or placebo (Jung *et al*., 2017).

In August 2016, the US FDA published ‘Dietary Supplements: New Dietary Ingredients Notifications and Related Issues: Guidance for Industry, Draft Guidance’ which will replace a draft guidance document issued in July 2011 (NDI Draft Guidance, 2016). According to this document, synthetic forms of substances that occur naturally cannot be used in dietary supplements in the USA, and as a consequence, synthetic *p*‐synephrine HCl cannot be used in dietary supplements.

It should be noted that synthetic derivatives of *p*‐synephrine as methylsynephrine HCl (oxilofrine HCl) are also prohibited from being used in dietary supplements, and the US FDA has sent out warning letters to manufacturers using methylsynephrine in products (Pawar and Grundel, 2016). In addition, although isopropyl‐norsynephrine (Wenkeova *et al*., 1975; Mercader *et al*., 2011) and *t*‐butyl‐norsynephrine (Wenkeova *et al*., 1976) have been shown to enhance lipolysis in human adipose tissues, these synthetic derivatives are also prohibited from being used in dietary supplements.

*p*‐Synephrine should not be confused with methylsynephrine, which has been reported as an adulterant in dietary supplements (Vaysse *et al*., 2010; Cohen *et al*., 2016). While the reviewed studies in the preceding texts indicate that *p‐*synephrine acts as a nonstimulatory thermogenic, two studies have examined the effects of a multicomponent product that contained methylsynephrine HCl but no *p*‐synephrine (Bloomer *et al*., 2009a, 2009b). Significant increases in heart rate as well as systolic and diastolic blood pressure occurred in both studies in response to the consumption of the product. These results indicate that methylsynephrine, which has a phenylpropylamine structure more closely related to ephedrine (Rasmussen and Keizers, 2015), acts as a stimulant. Again, it should be emphasized that synthetic *p*‐synephrine HCL and methylsynephrine as well as synthetic esters of norsynephrine are prohibited by the US FDA from being used in dietary supplements.

In addition to the human studies, tens of millions of doses of bitter orange extract (Advantra Z^®^; *p*‐synephrine)‐containing products have been consumed in the USA as well as internationally by millions of individuals without the report of serious incidents. Furthermore, a variety of orange juices (i.e., Mandarins, Marrs sweet oranges, and clementines) contain up to 20–25 mg of *p*‐synephrine per 8‐oz glass (Dragull *et al*., 2008; Uckoo *et al*., 2010). Thus, on a daily basis, millions of individuals consume *p*‐synephrine in the form of juices and orange‐related food products as marmalades alone and in combination with caffeine from various beverages without adverse events. No serious adverse events have ever been directly attributable to bitter orange and *p*‐synephrine, in spite of the extensive use in various forms of products.

## Animal Studies

Several animal studies have been conducted by the National Center for Toxicological Research in conjunction with the US FDA regarding the safety of bitter orange extract and *p*‐synephrine (Hansen *et al*., 2011, 2012, 2013). In a study that examined the developmental toxicity of C. aurantium in rats, the authors concluded that doses of up to 100 mg of *p*‐synephrine/kg body weight did not produce developmental toxicity (Hansen *et al*., 2011). At this dose, there were no adverse effects with respect to fetal weight, incidence of gross, skeletal or visceral abnormalities, or embryo lethality. In an 80‐kg (176 lb) human, a dose of 100 mg/kg in rats is equivalent to a *p*‐synephrine dose of approximately 1.3 g, an amount that is 26 times greater than a typical 50‐mg human dose.

These authors examined the physiological effects of administering *p*‐synephrine in the form of bitter orange extract as well as isolated *p‐*synephrine to rats for 28 days at doses of up to 50 mg/kg with and without 25 mg caffeine/kg (Hansen *et al*., 2012). Minimal, clinically insignificant effects were produced by these high doses of *p*‐synephrine with respect to heart rate and blood pressure. As expected, caffeine alone and in combination with *p*‐synephrine produced more pronounced but small increases in heart rate and blood pressure. The 25‐mg/kg dose of caffeine in the rats is equivalent to approximately a 325‐mg dose in an 80‐kg human.

The potential cardiovascular effects of bitter orange extract and *p*‐synephrine were also examined in exercised rats given up to 50‐mg/kg *p‐*synephrine in the presence and absence of 25‐mg/kg caffeine for 28 days (Hansen *et al*., 2013). Small increases in heart rate and body temperature were reported due to caffeine, while *p*‐synephrine exhibited small, clinically insignificant effects on blood pressure at the high dose. The dose of 50‐mg/kg *p*‐synephrine is approximately 13 times higher than a typical 50‐mg dose in an 80‐kg (176 lb) human in conjunction with a dose of approximately 325‐mg caffeine. The adrenergic receptor binding of *p*‐synephrine in rodents is as much as ten‐fold greater than in humans (Carpene' *et al*., 1999, 2014; Mercader *et al*., 2011), which can readily account for the observed, small cardiovascular effects in rodents (Hansen *et al*., 2012, 2013; Verpeut *et al*., 2013) when no such effects are observed in humans (Stohs *et al*., 2012; Stohs and Shara, 2013; Kaats *et al*., 2013; Shara *et al*., 2016).

In a study involving high fat diet‐induced obese rats, the animals were given bitter orange extract (5.6 mg/kg) for 10 days orally (Verpeut *et al*., 2013). The bitter orange extract had no effect on body weight or food intake but did result in a 4% increase in heart rate, with significant decreases in plasma corticosterone and norepinephrine levels as well as a decrease in frontal cortex dopamine levels. No effect on epinephrine levels was observed. Similar hormonal effects have not been observed in humans or reported in any other animal studies. The bitter orange extract contained 6% *p*‐synephrine, and therefore, the dosing was 0.34‐mg *p*‐synephrine/kg. The dose in this rat study is equivalent to 4.4‐mg *p*‐synephrine given to an 80‐kg (176 lb) human, an amount that is three‐fold to five‐fold less than is consumed daily in an 8‐oz glass of various orange juices (Dragull *et al*., 2008; Uckoo *et al*., 2010), and is a dose that would not be expected to produce measurable effects in humans.

In another study, mice were treated daily with bitter orange extract (7.5% *p*‐synephrine) at doses of 400, 2000, or 4000 mg/kg (corresponding to 30, 150, and 300 mg of *p*‐synephrine/kg) or with 30 mg or 300 mg of *p*‐synephrine/kg (Arbo *et al*., 2009). The 300‐mg/kg dose is approximately 78 times a typical 50‐mg human dose of *p*‐synephrine. A reduction in body weight gain was observed at all doses relative to controls. No adverse effects were observed regarding organ weights, biochemical parameters, blood pressure, or heart rate in the treated mice at any of the doses.

In addition, both doses of *p*‐synephrine and the high dose of the bitter orange extract resulted in increases in the antioxidant and tissue protectant glutathione. The bitter orange extract decreased malondialdehyde content (an indicator of lipid peroxidation and lipid damage), and *p*‐synephrine increased catalase, which neutralizes hydrogen peroxide (Arbo *et al*., 2009). The results indicated a beneficial effect for both the bitter orange extract and *p‐*synephrine with respect to weight loss without adverse effects while also providing an antioxidant and tissue protective effect.

Several unpublished safety studies have also been conducted on the safety of *p*‐synephrine (personal correspondence). A single oral dose of 10 000 mg/kg of a 6% *p*‐synephrine containing bitter orange extract did not cause lethality in rats. Furthermore, 5000 mg/kg of a 50% *p*‐synephrine containing extract (Advantra Z^®^) administered orally did not produce deaths in rats, indicating that the LD50 of this extract was greater than 5000 mg/kg. For the sake of comparison, it should be noted that the oral LD50 of sodium chloride, common table salt, is about 3000 mg/kg. The oral administration of a 50% *p*‐synephrine bitter orange extract at a dose of 2000 mg/kg to female rats for four consecutive days did not have any toxicological effects (Deshmukh *et al*., 2017a), while a dose of 1000 mg/kg for 90 days produced no deaths or serious adverse effects (Deshmukh *et al*., 2017b). The animals exhibited burrowing of the heads in the bedding material and piloerection, which disappeared by the end of the study.

In summary, these animal studies indicate that at the doses of *p*‐synephrine commonly used in dietary supplements, either alone or in conjunction with commonly used doses of caffeine, cardiovascular, or other adverse effects would not be expected and support the results of the growing number of human studies that have been conducted to date.

## Mutagenicity Studies

The potential mutagenicity of a bitter orange extract (Advantra Z^®^) containing 50% *p*‐synephrine was assessed by using the bacterial *Salmonella typhimurium* reverse mutation assay (Ames Test; Deshmukh *et al*., 2017a). The assay was performed by the preincubation method by using the tester strains TA1535, TA97a, TA98, TA100, and TA102, each of which contains a different kind of mutation in the histidine operon. The test was conducted in duplicate in the presence and absence of an S9 metabolic activation system. The results indicated that the bitter orange extract (50% *p*‐synephrine) did not induce cytotoxic effects in the tester strains at and up to a concentration of 5000 μg/plate in the presence and absence of metabolic activation. It was therefore concluded that this standardized bitter orange extract is nonmutagenic in the *S. typhimurium* reverse mutation assay (Ames test). The reproducibility of the negative control results was confirmed by repeating the experiment.

The results of this mutagenicity study are also supported by several additional studies. Morimoto *et al*. (1982) conducted a mutagenicity screening of the aqueous and methanolic extracts of 104 crude drugs, including C. aurantium (bitter orange). The assays involved the *Bacillus subtilis* rec‐assay by using *B. subtilis* strains H17 REC^+^ and M45 REC^−^, and the *S. typhimurium* microsomal reversion assay by using strains TA98 and TA100. The bitter orange extracts were shown to be negative in both assays, although large numbers of the extracts of other crude drugs were shown to be positive in both assays.

In another study published only in thesis form (Kaefer, 2014), *p*‐synephrine and its forced degradation products were studied for their potential mutagenic effects. Degradation of *p*‐synephrine was produced under acidic, basic, oxidative, and proteolytic conditions. The results indicated that *p*‐synephrine and its degradation products were nonmutagenic. *p*‐Synephrine did not cause micronucleus production of cultured cells up to the highest tested concentration of 100 ng/mL nor did it affect the viability or proliferation of leukocytes at this concentration. Under the experimental conditions employed and using the DNA comet tail assay, a small increase in DNA damage was observed at 100 ng/mL, a concentration that is about 10 times greater than the blood levels observed in humans 2 h after a 49‐mg dose of *p*‐synephrine (Shara *et al*., 2016). No effect on DNA was observed at a *p*‐synephrine concentration of 50 ng/mL.

## Mechanistic Studies

Cardiovascular effects of ligands are associated with adrenergic receptor binding. In general, vasoconstriction occurs when ligands bind to *α*‐adrenergic receptors, while binding to β‐1 adrenergic receptors results in cardiovascular contractility and increased heart rate. Ligand binding to β‐2 adrenergic receptors is associated with bronchodilation (Inchiosa, 2011). Human and animal studies that were reviewed in the preceding texts indicate that adverse cardiovascular effects are not commonly associated with *p*‐synephrine, although they are widely known to be associated with the consumption of ephedrine and ephedra products (Haller and Benowitz, 2000). The lack of blood pressure and heart rate effects in conjunction with *p*‐synephrine is believed to be due to the fact that *p*‐synephrine binds much more poorly to α‐1, α‐2, β‐1, and β‐2 adrenergic receptors than other ligands as norepinephrine, epinephrine, ephedrine, and *m‐*synephrine (phenylephrine; Stohs *et al*., 2011b; Stohs and Badmaev, 2016).

Brown *et al*. (1988) observed that *p*‐synephrine was 1000‐fold less active in binding to α‐1 and α‐2 adrenergic receptors than norepinephrine while binding of the synthetic compound *m*‐synephrine to these two receptors was 150‐fold and 6‐fold less, respectively, than norepinephrine. Ma *et al*. (2010) concluded that *p*‐synephrine acts as an antagonist rather than an agonist with respect to human α‐2a adrenergic and α‐2c adrenergic receptors. Several studies have concluded that the hydroxyl group in the para position of the ring as occurs in *p*‐synephrine decreases adrenergic receptor binding and the subsequent cardiovascular effects (Mukherjee *et al*., 1976; Ma *et al*., 2010). Jordan *et al*. (1987) concluded that *p*‐synephrine bound to the β‐1 and β‐2 adrenergic receptor about 10 000‐fold or less actively than norepinephrine.

Various studies have shown that *p*‐synephrine binds to β‐3 adrenergic receptors, resulting in an increase in the body's ability to breakdown fats (Carpene' *et al*., 1999, 2014; Mercader *et al*., 2011). Binding to β‐3 adrenergic receptors does not influence heart rate or blood pressure. Because *p*‐synephrine exhibits little or no binding to α‐1, α‐2, β‐1, and β‐2 adrenergic receptors, cardiovascular effects as an increase in heart rate and blood pressure are not experienced at commonly used doses of *p*‐synephrine, unlike a number of other phenylethylamine and phenylpropylamine derivatives. It should again be noted that *p*‐synephrine binds much more readily to adrenergic receptors from rodents than humans, and therefore, caution is required in extrapolating animal studies to humans (Carpene *et al*., 1999, 2014; Mercader *et al*., 2011).

Carpene *et al*. (2014) also showed that, in human and rat adipocytes tyramine and *N*‐methyltyramine, minor components found in bitter orange extract inhibited lipolysis in contrast to *p‐*synephrine. A review of the adrenergic receptor binding and effects of *N*‐methyltyramine indicate that it acts as an α‐adrenergic receptor antagonist while promoting appetite and inhibiting lipolysis, effects counter to commonly perceived ideas regarding its use in dietary supplements (Stohs and Hartman, 2015).

Several studies have examined the effects of *p*‐synephrine and bitter orange extracts on carbohydrate metabolism in perfused rat liver. Peixoto *et al*. (2012) demonstrated that both *p*‐synephrine and bitter orange extract increased glycogenolysis, glycolysis, oxygen uptake, glucose output, and perfusion pressure. At low concentrations, bitter orange extract tended to promote gluconeogenesis, but at high concentrations, it was inhibitory. These changes were partially sensitive to α‐adrenergic and β‐adrenergic antagonists. The concentrations of the bitter orange extract that contained 8.2% *p*‐synephrine ranged from 100 to 400 mg/L in the perfusate, while the results for 200‐μM *p*‐synephrine were reported. These authors concluded that most of the actions of bitter orange extract and *p*‐synephrine are catabolic and compatible with weight‐loss effects, which are a common application.

In a similar study, the perfusion of rat liver with *p*‐synephrine was shown to significantly stimulate glycogenolysis, glycolysis, gluconeogenesis, oxygen uptake, portal perfusion pressure, and the cytosolic redox state of the NAD(+)/NADH couple (de Oliveira *et al*., 2014). A Ca(2+) and cyclic AMP (cAMP) dependency was found for these effects. These effects were shown to be at least in part mediated by α‐adrenergic and β‐adrenergic signaling while requiring the simultaneous participation of both cAMP and Ca(2+). The perfusate concentrations of *p*‐synephrine ranged from 10 to 500 μM, with measurable effects being obtained at the lowest concentration. A recent study has shown that the blood serum concentration of *p*‐synephrine following a 49‐mg oral dose was approximately 10 ng/mL (0.06 μM) 2 h post ingestion (Shara *et al*., 2016). How this serum concentration of *p*‐synephrine following oral ingestion relates to the concentrations in the study in the preceding texts required to affect carbohydrate metabolism in the liver is not clear.

It has been shown that *p*‐synephrine suppresses appetite and enhances eating control in humans (Kaats and Stohs, 2017) and animals (Arbo *et al*., 2009). Neuromedin U2 receptor (NMUR2) is present in the hypothalamic regions of the brain and is associated with regulation of food intake, energy balance, stress, and nociception (Zheng *et al*., 2014). In a study involving the use of NMUR2 negative and short hairpin RNA knockdown HEK293 cell lines, *p*‐synephrine was shown to bind to this receptor with high efficacy and potency. Concentrations used in this study ranged from 0.001 to 1000 μM, with an effect detected at a concentration as low as 0.01 μM, a concentration within the range in serum produced by a single, typical 49‐mg oral dose of *p*‐synephrine (Shara *et al*., 2016). The concentration values that produced efficacy, 50% maximum possible effect (EC50), and potency were 7.2, 6.6, and 0.227 μM, respectfully (Zheng *et al*., 2014). These results provide a possible and plausible mechanism for the reported appetite suppression and eating control that has been observed in both human and animal studies in conjunction with *p*‐synephrine and bitter orange extract. However, the ability and extent to which *p*‐synephrine can cross the blood brain barrier to achieve functional concentrations have not been specifically determined.

A study investigated the effect of *p*‐synephrine on glucose consumption and its mechanism of action in L6 skeletal muscle cells (Hong *et al*., 2012). Treatment of the cells with 0–100 μM *p*‐synephrine did not affect cell viability and increased basal glucose consumption by over 50% relative to control in a dose‐dependent manner. Their results further indicated that the consumption of glucose by *p*‐synephrine involved Glut4‐dependent glucose uptake that was in turn dependent upon *p*‐synephrine stimulation of AMP‐activated protein kinase phosphorylation, providing additional mechanistic information.

In a subsequent study, the effects of *p*‐synephrine on glucose production and lipid accumulation in H411E rat liver cells were investigated (Cui *et al*., 2014). *p*‐Synephrine dose‐dependently (0–100 μM) decreased glucose production, and α‐adrenergic and β‐adrenergic receptor antagonists did not interrupt *p*‐synephrine‐induced suppression of glucose production. *p*‐Synephrine failed to affect palmitic acid‐induced cytoplasmic accumulation. The results suggested that the effects of *p*‐synephrine on gluconeogenesis occurred independently of adrenergic receptor interactions. Studies by other investigators showed a bi‐phasic response with respect to glucose production in perfused rat livers, and catabolic effects outweighed anabolic activity (Peixoto *et al*., 2012; de Oliveira *et al*., 2014).

Several studies have examined the antiinflammatory activity of *p*‐synephrine*. p*‐Synephrine was shown to suppress lipopolysaccharide‐induced acute lung injury in mice by inhibiting the NF‐κB signaling pathway (Wu *et al*., 2014). In mice induced with lipopolysaccharide, *p*‐synephrine significantly reduced the amount of inflammatory cells in the lungs, decreased the levels of reactive species, enhanced superoxide dismutase activity, decreased tumor necrosis alpha and interleukin‐6, and increased interleukin‐10. The doses of *p*‐synephrine that were given to the mice were 5 and 15 mg/kg, which corresponds to doses of 32.4 and 97.3 mg for an 80‐kg human, doses that are within the range commonly used.

Eotaxin‐1 is a potent chemoattractant and mediator for eosinophils during development of eosinophilic inflammation. In studies in NIH/3T3 mouse fibroblasts and in normal human fibroblasts in culture, *p*‐synephrine dose‐dependently (10–300 μM) inhibited IL‐4‐induced eotaxin‐1 expression through the inhibition of signal transducer and activator of transcription (STAT6) phosphorylation that acts as a signal transducer immediately downstream from IL‐4 (Roh *et al*., 2014). STAT6 is critical in activating cytokine gene expression and cytokine signaling in immune and target tissue cells. *p*‐Synephrine also inhibited eosinophil recruitment induced by eotaxin‐1 overexpression. Of interest was the observation that the synthetic *m*‐synephrine (phenylephrine) had little effect on eotaxin‐1 induction, indicating that it had little antiinflammatory activity. These authors concluded that *p*‐synephrine exerts antiinflammatory effects at least in part by inhibiting eotaxin‐1 expression (Roh *et al*., 2014).

As previously discussed, Arbo *et al*. (2009) observed that both bitter orange extract and *p*‐synephrine exerted antioxidant and tissue protectant effects in mouse liver. In an *in vitro* study involving a rat adrenal pheochromocytoma (PC12) cell line, a C. aurantium (bitter orange) extract was shown to protect against glutamate cytotoxicity in these cells, significantly reducing lipid peroxidation (MDA production), reactive oxygen species production, and cell apoptosis (Hosseini *et al*., 2016). The authors concluded that the extract exhibited neuroprotective effects. Unfortunately, the chemical composition and *p*‐synephrine content of the bitter orange extract were not determined, and therefore, no dose related effects can be determined.

## Discussion

Bitter orange extracts and *p*‐synephrine have been used for weight loss/weight management, sports performance, appetite control, energy, and mental focus and cognition. Studies supporting their use exist with respect to weight management, sports performance, energy, and appetite control and are reviewed in the preceding texts. However, the number of studies demonstrating and supporting some of these properties is small and a need exists for additional studies.

Studies in humans indicate that the *p*‐synephrine has a wide margin of safety with an oral LD50 greater than 2500 mg/kg in rats. Approximately 30 human studies involving over 600 subjects have demonstrated that *p*‐synephrine does not produce cardiovascular effects at doses up to 100 mg. Approximately 45% of the subjects in these studies were overweight or obese and over 40% of the subjects consumed caffeine in conjunction with bitter orange extract (*p‐*synephrine). *p*‐Synephrine binds up to 10 times more readily to adrenergic receptors in rodents than humans (Carpene' *et al*., 1999, 2014; Mercader *et al*., 2011), which can explain small cardiovascular effects in some animal studies at very high doses (Hansen *et al*., 2012, 2013).

*p*‐Synephrine, the primary active constituent in bitter orange extracts, at least in part, exerts its effects through multiple mechanisms including its binding to β‐3 adrenergic receptors that regulate lipid and carbohydrate metabolism, NMUR2s, and AMP‐activated protein kinase, cAMP, and Ca(2+)‐dependent mechanisms (Hong *et al*., 2012; Stohs *et al*., 2011b; Zheng *et al*., 2014; de Oliveira *et al*., 2014). Because *p*‐synephrine exhibits little or no binding to α‐1, α‐2, β‐1, and β‐2 adrenergic receptors, *p*‐synephrine exerts metabolic enhancement without acting as a central nervous system or cardiovascular stimulant at commonly used doses and therefore does not increase heart rate or blood pressure. As a consequence, *p*‐synephrine functions as a nonstimulant thermogenic agent (Stohs and Badmaev, 2016) without altering blood chemistries, blood cell counts, ECGs, or other cardiovascular parameters (Stohs *et al*., 2012; Kaats *et al*., 2013; Shara *et al*., 2016). However, because low‐level binding does occur to α‐1 and β‐1 and β‐2 adrenergic receptors, some effects may occur in part through these receptors.

Various studies have also demonstrated the antiinflammatory effects of bitter orange extract and *p*‐synephrine in rodent and *in vitro* models. *p*‐Synephrine and bitter orange extracts have been shown to inhibit the NF‐κB signaling pathway and therefore downregulate tumor necrosis alpha and interleukin‐6 as well as reactive oxygen species production (Wu *et al*., 2014). A study has also shown that the antiinflammatory effects may also involve inhibition of eostaxin‐1 and the STAT6 signaling pathway (Roh *et al*., 2014). Therefore, multiple pathways and mechanisms of action may be involved.

In order for *p*‐synephrine to exert various physiological/pharmacological effects when given orally, adequate absorption must be achieved with appropriate blood and tissue levels. However, little information is available regarding absorption, metabolism, and tissue distribution. That absorption does occur and was demonstrated by Shara *et al*. (2016), who observed that blood levels of approximately 10 ng/mL (0.06 μM) were achieved 2 h after an oral dose of 49 mg of *p*‐synephrine. In *in vitro* cell culture studies, responses have been reported with concentrations of *p*‐synephrine as low as 0.01 μM (Zheng *et al*., 2014), although various perfusion studies have reported effects with concentrations as low as 10 μM (Peixoto *et al*., 2012; de Oliveira *et al*., 2014).

The *N*‐demethylated product of *p*‐synephrine is *p*‐octopamine. As previously noted, little or no *p*‐octopamine is present in bitter orange extracts (Pellati and Benvenuti, 2007; Stohs, 2015), contrary to popular belief. Although *p‐*synephrine undergoes rapid *N*‐demethylation to *p*‐octopamine, no *p*‐octopamine is detected in the urine at doses of up 150 mg of *p*‐synephrine orally due to rapid oxidative deamination of the *p*‐octopamine (Thevis *et al*., 2012; Medana *et al*., 2013). *p‐*Synephrine has been shown to be rapidly extracted from the blood by the liver. The single pass extraction of *p*‐synephrine was shown to be higher than 90% at a portal concentration of 10 μM (de Silva‐Pereira *et al*., 2016), indicating rapid removal and metabolism. The half‐life of *p*‐synephrine has been estimated to be in the range of 2–3 h (Hengtmann and Aulepp, 1978; Haller *et al*., 2005, 2008), agreeing with the blood levels observed by Shara *et al*. (2016) at 2 h post ingestion.

Grapefruit (*Citrus paradisi*) juice is known for its ability to alter drug metabolism through inhibition of cytochrome P450‐3A4 and results in drug–food interactions that may be life threatening (An *et al*., 2015). The primary active ingredients in *Citrus* responsible for these effects are the furanocoumarins bergapten, bergamottin, and 6′,7′‐dihydroxybergamottin (Guo *et al*., 2000; Paine *et al*., 2006; Fujita *et al*., 2008; Messer *et al*., 2011; Greenblatt *et al*., 2012). Studies have shown that Seville, mandarin, and clementine orange juices have the potential to produce drug interactions (see, for example, Abdelkawy *et al*., 2016; Theile *et al*., 2016), presumably due to their furanocoumarin content. Gurley *et al*. (2004) showed that, in humans receiving 30.6 mg of *p*‐synephrine per day in the form of a bitter orange (C. aurantium), no significant effects were observed on CYP3A4, CYP1A2, CYP2D6, or CYP2E1, the major drug‐metabolizing cytochrome enzymes.

Analysis of the furanocoumarin content of four bitter orange (Advantra Z^®^) extracts by liquid chromatography‐mass spectroscopy has shown that the total furanocoumarin content of each of these extracts was less than 20 μg/g, amounts insufficient to exert significant effects on the metabolism of susceptible drugs in human subjects at commonly used doses (Stohs *et al*., 2014). As a consequence, bitter orange extracts containing *p*‐synephrine with essentially no furanocoumarins would not be expected to produce potential drug interactions similar to grapefruit juice, and no significant interactions have been reported. Furthermore, the effects of *Citrus* juices including C. aurantium cannot be extrapolated to bitter orange extracts and *p*‐synephrine.

Flavonoids as naringin and hesperidin are widely distributed in *Citrus* species and are known to exhibit antioxidant, antiinflammatory, hepatoprotective, antineoplastic, thermogenic, and lipolytic effects (see, for example, Stohs *et al*., 2011c; Choi *et al*., 2015; Lee *et al*., 2015; Stohs and Badmaev, 2016; Chen *et al*., 2016). Fujita *et al*. (2008) conducted a comparative evaluation of the inhibitory activity of 12 immature *Citrus* fruit extracts against cytochrome P450 isoforms and demonstrated that, although furanocoumarins are primarily responsible for inhibition of cytochrome P450, flavonoids are a contributing factor. The flavonoid content of juice from C. aurantium is about one‐fifth the flavonoid content of grapefruit juice (Edwards and Bernier, 1996).

Okada *et al*. (2017) have shown that an ethanol extract of bitter orange (dried immature fruit) induced expression of cytochrome CYP3A4 and P‐glycoprotein in LS180 cells in culture by upregulating pregnane X receptor. Naringin and narirutin were the primary constituents of the extract. The amount of material required to produce an inductive effect was not stated, and therefore, it is not possible to extrapolate the results to the human consumption of a bitter orange extract.

The inhibitory constants for flavonoids involving human cytochrome P450 are in the range of 25 μM, which, for naringin, is about 6.8 μg/mL (Burkina *et al*., 2016), a concentration that is not attainable in plasma by the ingestion of a typical dose of bitter orange extract that contains 50% *p‐*synephrine. Analysis has shown that the level of flavonoids in a standardized bitter orange extract (i.e., Advantra Z^®^) is below 1% (unpublished). Therefore, in 100 mg of a 50% containing bitter orange extract, only about 1 mg of flavonoids would be present, an amount far too small to exert a significant effect on drug metabolism.

Finally, it has been recommended that *p*‐synephrine and bitter orange extract not be used under a variety of conditions (see, for example, Marles, 2011; Lynch, 2013; Natural Medicines Comprehensive Database, 2016). The majority of these contraindicated conditions are based on the assumption that *p*‐synephrine exhibits cardiovascular effects, and these warnings have been extrapolated from warnings that were associated with the use of ephedra products. The contraindicated conditions that have been proposed include individuals with high blood pressure, thyroid, kidney, liver, or heart disease; people with psychiatric or epileptic disorders; those using antihypertensive drugs, sympathomimetics, monoamine oxidase inhibitors, thyroid medications, and antidepressants; and children and women who are pregnant or breast feeding. No evidence exists to support these proposed contraindications.

It should be remembered that very large numbers of people consume *p*‐synephrine on a daily basis in the forms of citrus juices and foods as well as dietary supplements with no known or apparent adverse effects. There are no indications that *p*‐synephrine adversely affects the heart, liver, kidneys, or thyroid at doses up to 100 mg per day (Stohs *et al*., 2012; Kaats *et al*., 2013; Shara *et al*., 2016; Kaats and Stohs, 2017), and as a consequence, there is no evidence supporting the proposed contraindications. Furthermore, studies have shown that no teratogenicity (Hansen *et al*., 2011) or mutagenicity (Morimoto *et al*., 1982; Kaefer, 2014; Deshmukh *et al*., 2017a) occurs in response to *p*‐synephrine and bitter orange extracts. However, some cautionary statements regarding use of bitter orange extract and *p*‐synephrine may be warranted and are subject to further discussions.

The studies in the preceding texts indicate that *p*‐synephrine cannot be equated with ephedrine and the effects of ephedrine cannot be extrapolated to *p*‐synephrine due to structural differences, which greatly alter receptor binding characteristics, pharmacokinetic properties, and the pharmacological/physiological effects produced. At the doses commonly used, *p*‐synephrine does not product significant cardiovascular effects or other adverse events. Additional studies are required to determine at what dosage levels adverse effects might be expected to occur, and a human dose response study involving *p‐*synephrine and caffeine would be very informative.

## Conflict of Interest

The author has served as a consultant for Novel Ingredients, a company that markets bitter orange (Citrus aurantium) extracts.
